# *C*-Methyl­calix[4]resorcinarene–1,4-bis­(pyridin-3-yl)-2,3-diaza-1,3-butadiene (1/2)

**DOI:** 10.1107/S1600536811054717

**Published:** 2012-01-07

**Authors:** Konstantin A. Udachin, Md. Badruz Zaman, John A. Ripmeester

**Affiliations:** a Steacie Institute for Molecular Sciences, National Research Council of Canada, 100 Sussex, Ottawa, Ontario, Canada K1A 0R6; b Center of Excellence for Research in Engineering Materials, Faculty of Engineering, King Saud University, Riyadh 11421, Saudi Arabia

## Abstract

In the title compound, 2C_12_H_10_N_4_·C_32_H_32_O_8_, the calixarene adopts a rctt conformation with dihedral angles of 138.40 (1) and 9.10 (1)° between the opposite rings. The dihedral angles between the rings of the pyridine derivative are 8.80 (1) and 9.20 (1)°. In the crystal, adjacent *C*-methylcalix[4]resorcinarene molecules are connected into columns parallel to [010] by O—H⋯O hydrogen bonds. O—H⋯N hydrogen bonds between the axial phenoxyl groups and bipyridine molecules link the columns into sheets parallel to (011), which are connected by O—H⋯N hydrogen bonds. Further O—H⋯N hydrogen bonds link the bipyridine and *C*-methylcalix[4]resorcinarene molecules, giving rise to a three-dimensional network.

## Related literature

For the synthesis and structure of the 1,4-di-3-pyridyl-2,3-diaza-1,3-butadiene ligand, see: Ciurtin *et al.* (2001 ▶). For coordination polymers of 1,4-di-3-pyridyl-2,3-diaza-1,3-butadiene structures, see: Dong *et al.* (2004 ▶). For the structure of *C*-methyl­calix[4]resorcinarene, see: Kuzmicz *et al.* (2010 ▶). For *C*-methyl­calix[4]resorcinarene co-crystal structures, see: MacGillivray *et al.* (2001 ▶); Ma & Coppens (2004 ▶); Momose & Bosch (2010 ▶). For the stereochemistry of *C*-methyl­calix[4]resorcinarene, see: Moore & Matthews (2009 ▶).
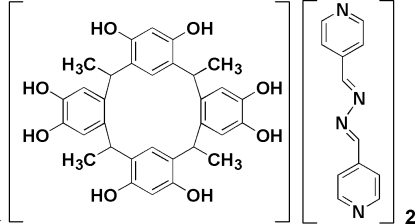


## Experimental

### 

#### Crystal data

2C_12_H_10_N_4_·C_32_H_32_O_8_
*M* *_r_* = 965.06
Monoclinic, 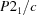

*a* = 12.2998 (10) Å
*b* = 26.232 (2) Å
*c* = 16.1097 (13) Å
β = 109.324 (2)°
*V* = 4904.9 (7) Å^3^
*Z* = 4
Mo *K*α radiation
μ = 0.09 mm^−1^
*T* = 173 K
0.35 × 0.20 × 0.15 mm

#### Data collection

Bruker Kappa APEX CCD diffractometer
Absorption correction: multi-scan (*SADABS*; Sheldrick, 1996 ▶) *T*_min_ = 0.970, *T*_max_ = 0.987
58266 measured reflections
12755 independent reflections
7952 reflections with *I* > 2σ(*I*)
*R*_int_ = 0.058

#### Refinement

*R*[*F* ^2^ > 2σ(*F* ^2^)] = 0.050
*wR*(*F* ^2^) = 0.131
*S* = 1.02
12755 reflections
656 parameters
H-atom parameters constrained
Δρ_max_ = 0.27 e Å^−3^
Δρ_min_ = −0.24 e Å^−3^

### 

Data collection: *SMART* (Bruker, 2003 ▶); cell refinement: *SAINT* (Bruker, 2003 ▶); data reduction: *SAINT*; program(s) used to solve structure: *SHELXS97* (Sheldrick, 2008 ▶); program(s) used to refine structure: *SHELXL97* (Sheldrick, 2008 ▶); molecular graphics: *ATOMS* (Dowty, 1999 ▶); software used to prepare material for publication: *SHELXL97*.

## Supplementary Material

Crystal structure: contains datablock(s) I, global. DOI: 10.1107/S1600536811054717/vm2143sup1.cif

Structure factors: contains datablock(s) I. DOI: 10.1107/S1600536811054717/vm2143Isup2.hkl

Additional supplementary materials:  crystallographic information; 3D view; checkCIF report

## Figures and Tables

**Table 1:** Hydrogen-bond geometry (Å, °)

*D*—H⋯*A*	*D*—H	H⋯*A*	*D*⋯*A*	*D*—H⋯*A*
O1—H1⋯N4*B*	0.84	1.95	2.765 (2)	162
O2—H2⋯N1*A*	0.84	1.87	2.6986 (17)	167
O3—H3⋯O2^i^	0.84	1.99	2.8297 (17)	172
O4—H4⋯O5	0.84	2.15	2.9265 (17)	153
O5—H5⋯N4*A*^ii^	0.84	1.90	2.7338 (19)	175
O6—H6⋯N1*B*^ii^	0.84	1.95	2.7855 (19)	176
O7—H7⋯O6	0.84	2.14	2.9528 (18)	163
O8—H8⋯N2*B*^iii^	0.84	2.11	2.947 (2)	174

Symmetry codes: (i) 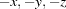; (ii) 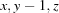; (iii) 
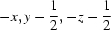
.

**Fig. 1.**
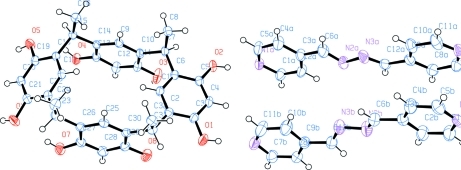
Molecular structure and atom naming scheme for the co-crystals (C-methylcalix[4]resorcinarene).2(1,4-di-3-pyridyl-2,3-diaza-1,3-butadiene). Displacement ellipsoids are drawn at the 50% probability level.

**Fig. 2.**
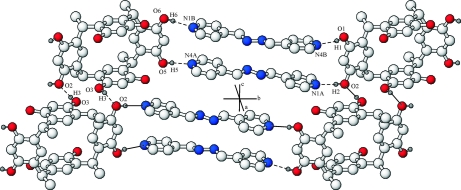
Packing diagram of co-crystals of (C-methylcalix[4]resorcinarene). 2(1,4-di-3-pyridyl-2,3-diaza-1,3-butadiene) viewed down the b axis showing linear one-dimentional hydrogen-bonding chains associations as dashed lines.

## supplementary crystallographic information

### Comment

C-Methylcalix[4]resorcinarene is the simplest member of the resorcinarene compounds and assemblies by means of intermolecular hydrogen bonding (Kuzmicz *et al.*, 2010). *C*-Methylcalix[4]resorcinarene has typically been crystallized with 4,4'-bipyridine type ligands to form solids with large cavities capable of including organic or inorganic guests (MacGillivray *et al.*, 2001; Ma & Coppens, 2004; Momose & Bosch, 2010). We used bidentate Schiff-base ligands 1,4-di-3-pyridyl-2,3-diaza-1,3-butadiene (Dong *et al.*, 2004) with *C*-methylcalix[4]resorcinarene and crystallized single-crystals using ethanol as solvent (Fig. 1).

Nearby *C*-methylcalix[4]resorcinarene molecules are connected into infinite columns parallel to the crystallographic [010] direction through two, center-of-symmetry-related, phenoxyl O–H···O hydrogen bonds per molecular pair [O3···O2 = 2.8297 (17) Å, O3–H3···O2 = 172.4 °]. As shown in Figure 2, O–H···N hydrogen bonds between the axial phenoxyl groups and bipyridine molecules link the columns into stair-like sheets parallel to the (011) plane [O2···N1A = 2.6986 (17)Å, O2–H2···N1A = 167.3 °; O1···N4B = 2.765 (2) Å, O1–H1···N4B = 162.3 °]. The sheets are connected with each other by a second set of bipyridylmolecules through O–H···N hydrogen bonds [O5···N4A = 2.7338 (19) Å, O5–H5···N4A = 174.9 °; O6···N1B = 2.7855 (19) Å, O6–H6···N1B = 175.9 °]. Additionally, zigzag –CH=N—N=CH– bridge of the bipyridine molecules and axial phenoxyl groups of *C*-methylcalix[4]resorcinarene molecules are also connected *via* O–H···N hydrogen bonds [O8···N2B = 2.947 (2) Å, O8–H8···N2B = 173.8 °] giving rise to a three-dimensional network.

Confirmation of calix[4]resorcinarene topology has been classified into four diffrent structures *i.e.* rccc (cone/crown), rcct (chair), rctt (diamond), rccc (boat) (Moore & Matthews, 2009). In the title compound, a pair of aromatic rings are almost coplanar, whereas the others are orthogonal at an angle of 88.1 and 95.7 ° from the plane facing the side-chains. This constructs an rctt conformation (Fig. 1).

### Experimental

*C*-methylcalix[4]resorcinarene (0.05 mmol) and 1,4-di-3-pyridyl-2,3-diaza-1,3-butadiene (0.05 mmol) were dissolved in 3 ml and 1.5 ml of ethanol, respectively. After that, the mixture was heated to 100 ° C for 4 h in an oven, cooled to room temperature at a rate of 20 ° C per hour and kept few days at room temperature. Colorless, plate-like crystals were collected after 2/3 days.

### Refinement

All hydrogen atoms were placed in calculated positions with C—H distances 0.95 Å (aryl), 0.98 Å (methyl), 1.00 Å (methine) and O—H distances 0.84 Å. All hydrogen *U*_eq_ were fixed at 1.2 times of the *U*_eq_ preceding nonhydrogen atom.

### Crystal data

**Table:**

2C_12_H_10_N_4_·C_32_H_32_O_8_	*F*(000) = 2032
*M**_r_* = 965.06	*D*_x_ = 1.307 Mg m^−^^3^
Monoclinic, *P*2_1_/*c*	Mo *K*α radiation, λ = 0.71070 Å
Hall symbol: -P 2ybc	Cell parameters from 280 reflections
*a* = 12.2998 (10) Å	θ = 5–30°
*b* = 26.232 (2) Å	µ = 0.09 mm^−^^1^
*c* = 16.1097 (13) Å	*T* = 173 K
β = 109.324 (2)°	Block, yellow
*V* = 4904.9 (7) Å^3^	0.35 × 0.20 × 0.15 mm
*Z* = 4	

### Data collection

**Table:**

Bruker Kappa APEX CCD diffractometer	12755 independent reflections
Radiation source: fine-focus sealed tube	7952 reflections with *I* > 2σ(*I*)
graphite	*R*_int_ = 0.058
φ and ω scans	θ_max_ = 28.8°, θ_min_ = 1.6°
Absorption correction: multi-scan (*SADABS*; Sheldrick, 1996)	*h* = −16→16
*T*_min_ = 0.970, *T*_max_ = 0.987	*k* = −35→35
58266 measured reflections	*l* = −21→21

### Refinement

**Table:**

Refinement on *F*^2^	Primary atom site location: structure-invariant direct methods
Least-squares matrix: full	Secondary atom site location: difference Fourier map
*R*[*F*^2^ > 2σ(*F*^2^)] = 0.050	Hydrogen site location: inferred from neighbouring sites
*wR*(*F*^2^) = 0.131	H-atom parameters constrained
*S* = 1.02	*w* = 1/[σ^2^(*F*_o_^2^) + (0.0601*P*)^2^ + 0.3967*P*] where *P* = (*F*_o_^2^ + 2*F*_c_^2^)/3
12755 reflections	(Δ/σ)_max_ < 0.001
656 parameters	Δρ_max_ = 0.27 e Å^−^^3^
0 restraints	Δρ_min_ = −0.24 e Å^−^^3^

### Special details

**Table:**

Geometry. All e.s.d.'s (except the e.s.d. in the dihedral angle between two l.s. planes) are estimated using the full covariance matrix. The cell e.s.d.'s are taken into account individually in the estimation of e.s.d.'s in distances, angles and torsion angles; correlations between e.s.d.'s in cell parameters are only used when they are defined by crystal symmetry. An approximate (isotropic) treatment of cell e.s.d.'s is used for estimating e.s.d.'s involving l.s. planes.
Refinement. Refinement of *F*^2^ against ALL reflections. The weighted *R*-factor *wR* and goodness of fit *S* are based on *F*^2^, conventional *R*-factors *R* are based on *F*, with *F* set to zero for negative *F*^2^. The threshold expression of *F*^2^ > σ(*F*^2^) is used only for calculating *R*-factors(gt) *etc*. and is not relevant to the choice of reflections for refinement. *R*-factors based on *F*^2^ are statistically about twice as large as those based on *F*, and *R*- factors based on ALL data will be even larger.

### Fractional atomic coordinates and isotropic or equivalent isotropic displacement parameters (Å^2^)

**Table:**

	*x*	*y*	*z*	*U*_iso_*/*U*_eq_	
O1	0.11346 (13)	0.10695 (5)	−0.29351 (8)	0.0452 (3)	
H1	0.0785	0.1339	−0.2898	0.054*	
O2	0.14016 (10)	0.09443 (4)	0.01170 (8)	0.0317 (3)	
H2	0.1311	0.1262	0.0079	0.038*	
O3	0.06394 (11)	−0.04247 (5)	−0.00563 (10)	0.0460 (3)	
H3	0.0035	−0.0591	−0.0118	0.055*	
O4	0.21608 (10)	−0.21043 (4)	0.02754 (8)	0.0331 (3)	
H4	0.2786	−0.2265	0.0467	0.040*	
O5	0.40391 (11)	−0.28018 (4)	0.03273 (7)	0.0354 (3)	
H5	0.3820	−0.3103	0.0188	0.042*	
O6	0.33853 (11)	−0.27263 (4)	−0.27806 (7)	0.0363 (3)	
H6	0.3226	−0.3034	−0.2735	0.054*	
O7	0.15451 (11)	−0.19612 (4)	−0.34688 (9)	0.0405 (3)	
H7	0.2145	−0.2140	−0.3330	0.049*	
O8	0.02134 (10)	−0.02744 (5)	−0.33701 (9)	0.0409 (3)	
H8	−0.0220	−0.0375	−0.3095	0.049*	
C1	0.24240 (14)	0.01166 (6)	−0.12715 (10)	0.0267 (3)	
H1A	0.2817	−0.0199	−0.1227	0.032*	
C2	0.20718 (14)	0.03655 (6)	−0.20799 (10)	0.0273 (3)	
C3	0.14939 (14)	0.08289 (6)	−0.21349 (11)	0.0295 (4)	
C4	0.12804 (14)	0.10278 (6)	−0.14037 (11)	0.0299 (4)	
H4A	0.0886	0.1343	−0.1447	0.036*	
C5	0.16409 (13)	0.07667 (6)	−0.06111 (11)	0.0257 (3)	
C6	0.22321 (13)	0.03049 (6)	−0.05246 (10)	0.0251 (3)	
C7	0.27180 (15)	0.00482 (6)	0.03648 (10)	0.0289 (4)	
H7A	0.2234	0.0161	0.0722	0.035*	
C8	0.39287 (17)	0.02532 (6)	0.08243 (12)	0.0403 (4)	
H8A	0.4437	0.0148	0.0498	0.048*	
H8B	0.4223	0.0117	0.1424	0.048*	
H8C	0.3905	0.0626	0.0846	0.048*	
C9	0.35759 (14)	−0.08547 (6)	0.04637 (10)	0.0258 (3)	
H9	0.4319	−0.0709	0.0586	0.031*	
C10	0.26378 (14)	−0.05285 (6)	0.03113 (10)	0.0263 (3)	
C11	0.15494 (15)	−0.07511 (6)	0.01108 (11)	0.0311 (4)	
C12	0.14228 (15)	−0.12767 (6)	0.01046 (11)	0.0319 (4)	
H12	0.0679	−0.1423	−0.0022	0.038*	
C13	0.23827 (14)	−0.15883 (6)	0.02831 (10)	0.0267 (3)	
C14	0.34808 (14)	−0.13859 (6)	0.04461 (10)	0.0241 (3)	
C15	0.44978 (13)	−0.17430 (6)	0.05513 (10)	0.0254 (3)	
H15	0.4489	−0.2006	0.0999	0.031*	
C16	0.56767 (15)	−0.14822 (7)	0.08735 (11)	0.0340 (4)	
H16A	0.6282	−0.1736	0.0927	0.041*	
H16B	0.5792	−0.1325	0.1448	0.041*	
H16C	0.5711	−0.1219	0.0451	0.041*	
C17	0.42627 (13)	−0.17562 (6)	−0.10809 (10)	0.0243 (3)	
H17	0.4426	−0.1401	−0.1034	0.029*	
C18	0.42989 (13)	−0.20196 (6)	−0.03196 (10)	0.0241 (3)	
C19	0.40501 (14)	−0.25408 (6)	−0.04049 (10)	0.0257 (3)	
C20	0.37761 (14)	−0.27811 (6)	−0.12184 (10)	0.0277 (3)	
H20	0.3620	−0.3137	−0.1265	0.033*	
C21	0.37305 (14)	−0.25029 (6)	−0.19626 (10)	0.0276 (3)	
C22	0.40007 (13)	−0.19834 (6)	−0.19061 (10)	0.0244 (3)	
C23	0.39568 (14)	−0.16694 (6)	−0.27175 (10)	0.0266 (3)	
H23	0.3775	−0.1911	−0.3225	0.032*	
C24	0.51371 (15)	−0.14370 (7)	−0.26115 (12)	0.0332 (4)	
H24A	0.5091	−0.1239	−0.3138	0.040*	
H24B	0.5706	−0.1710	−0.2533	0.040*	
H24C	0.5370	−0.1213	−0.2095	0.040*	
C25	0.31351 (14)	−0.07589 (6)	−0.28032 (10)	0.0263 (3)	
H25	0.3901	−0.0631	−0.2603	0.032*	
C26	0.29752 (14)	−0.12852 (6)	−0.29279 (10)	0.0261 (3)	
C27	0.18376 (15)	−0.14597 (6)	−0.32558 (11)	0.0298 (4)	
C28	0.09198 (15)	−0.11258 (7)	−0.34000 (11)	0.0336 (4)	
H28	0.0152	−0.1250	−0.3616	0.040*	
C29	0.11217 (14)	−0.06108 (6)	−0.32294 (11)	0.0302 (4)	
C30	0.22337 (14)	−0.04121 (6)	−0.29563 (10)	0.0259 (3)	
C31	0.23665 (14)	0.01636 (6)	−0.28613 (10)	0.0279 (3)	
H31	0.1798	0.0316	−0.3400	0.033*	
C32	0.35513 (15)	0.03607 (7)	−0.28153 (11)	0.0328 (4)	
H32A	0.3548	0.0734	−0.2812	0.039*	
H32B	0.3736	0.0239	−0.3328	0.039*	
H32C	0.4131	0.0236	−0.2276	0.039*	
N1A	0.14443 (13)	0.19726 (5)	0.01693 (10)	0.0358 (3)	
N2A	0.21252 (14)	0.38510 (6)	−0.00459 (11)	0.0439 (4)	
N3A	0.27335 (15)	0.43125 (6)	−0.00072 (11)	0.0439 (4)	
N4A	0.32909 (19)	0.62157 (6)	−0.00347 (11)	0.0517 (5)	
C1A	0.07431 (16)	0.23736 (7)	−0.00830 (13)	0.0408 (4)	
H1A1	−0.0064	0.2314	−0.0295	0.049*	
C2A	0.11289 (16)	0.28689 (7)	−0.00523 (14)	0.0422 (5)	
H2A	0.0598	0.3143	−0.0230	0.051*	
C3A	0.23069 (16)	0.29589 (7)	0.02429 (13)	0.0380 (4)	
C4A	0.30385 (17)	0.25468 (7)	0.05267 (14)	0.0468 (5)	
H4A1	0.3849	0.2594	0.0751	0.056*	
C5A	0.25681 (16)	0.20659 (7)	0.04773 (14)	0.0436 (5)	
H5A	0.3077	0.1785	0.0675	0.052*	
C6A	0.27847 (18)	0.34750 (7)	0.02479 (14)	0.0447 (5)	
H6A	0.3595	0.3526	0.0474	0.054*	
C7A	0.2177 (2)	0.61136 (8)	−0.02188 (14)	0.0559 (6)	
H7A1	0.1653	0.6391	−0.0325	0.067*	
C8A	0.1737 (2)	0.56213 (7)	−0.02647 (13)	0.0488 (5)	
H8A1	0.0932	0.5566	−0.0410	0.059*	
C9A	0.24915 (18)	0.52175 (7)	−0.00953 (11)	0.0393 (4)	
C10A	0.36571 (19)	0.53196 (7)	0.00988 (13)	0.0436 (5)	
H10A	0.4203	0.5050	0.0219	0.052*	
C11A	0.4010 (2)	0.58211 (7)	0.01135 (14)	0.0500 (5)	
H11A	0.4807	0.5887	0.0236	0.060*	
C12A	0.20629 (18)	0.46912 (7)	−0.01125 (11)	0.0393 (4)	
H12A	0.1268	0.4635	−0.0204	0.047*	
N1B	0.27941 (14)	0.62678 (6)	−0.25762 (10)	0.0405 (4)	
N2B	0.14281 (15)	0.44397 (6)	−0.25308 (12)	0.0502 (4)	
N3B	0.15395 (15)	0.39050 (6)	−0.25003 (12)	0.0483 (4)	
N4B	0.03932 (16)	0.20584 (6)	−0.28645 (12)	0.0506 (4)	
C1B	0.17541 (19)	0.60616 (8)	−0.27661 (16)	0.0539 (5)	
H1B	0.1106	0.6283	−0.2945	0.065*	
C2B	0.15533 (18)	0.55428 (8)	−0.27192 (15)	0.0501 (5)	
H2B	0.0790	0.5415	−0.2870	0.060*	
C3B	0.24791 (15)	0.52183 (7)	−0.24516 (11)	0.0339 (4)	
C4B	0.35582 (17)	0.54304 (8)	−0.22410 (14)	0.0455 (5)	
H4B	0.4222	0.5219	−0.2045	0.055*	
C5B	0.36766 (18)	0.59495 (8)	−0.23147 (13)	0.0456 (5)	
H5B	0.4432	0.6086	−0.2170	0.055*	
C6B	0.23676 (17)	0.46610 (7)	−0.24210 (12)	0.0395 (4)	
H6B	0.3041	0.4459	−0.2313	0.047*	
C7B	−0.05398 (19)	0.23441 (7)	−0.31747 (13)	0.0461 (5)	
H7B	−0.1265	0.2178	−0.3401	0.055*	
C8B	−0.05192 (17)	0.28715 (7)	−0.31876 (13)	0.0419 (4)	
H8B1	−0.1214	0.3060	−0.3417	0.050*	
C9B	0.05244 (17)	0.31199 (7)	−0.28629 (12)	0.0390 (4)	
C10B	0.15124 (19)	0.28258 (8)	−0.25297 (16)	0.0556 (6)	
H10B	0.2248	0.2981	−0.2293	0.067*	
C11B	0.1400 (2)	0.23020 (8)	−0.25510 (17)	0.0625 (6)	
H11B	0.2080	0.2103	−0.2329	0.075*	
C12B	0.05814 (18)	0.36805 (7)	−0.28603 (13)	0.0423 (4)	
H12B	−0.0097	0.3875	−0.3127	0.051*	

### Atomic displacement parameters (Å^2^)

**Table:**

	*U*^11^	*U*^22^	*U*^33^	*U*^12^	*U*^13^	*U*^23^
O1	0.0656 (9)	0.0323 (7)	0.0369 (7)	0.0184 (6)	0.0157 (6)	0.0106 (5)
O2	0.0408 (7)	0.0189 (5)	0.0403 (7)	0.0030 (5)	0.0202 (6)	−0.0016 (5)
O3	0.0388 (7)	0.0284 (6)	0.0798 (10)	0.0093 (6)	0.0316 (7)	0.0106 (7)
O4	0.0359 (7)	0.0212 (6)	0.0434 (7)	0.0006 (5)	0.0146 (6)	0.0036 (5)
O5	0.0568 (8)	0.0202 (6)	0.0310 (6)	−0.0032 (5)	0.0169 (6)	0.0010 (5)
O6	0.0541 (8)	0.0264 (6)	0.0296 (6)	−0.0084 (5)	0.0156 (6)	−0.0074 (5)
O7	0.0345 (7)	0.0301 (6)	0.0522 (8)	−0.0040 (5)	0.0079 (6)	−0.0088 (6)
O8	0.0293 (7)	0.0399 (7)	0.0523 (8)	0.0054 (5)	0.0117 (6)	0.0030 (6)
C1	0.0295 (8)	0.0205 (7)	0.0301 (8)	0.0030 (6)	0.0097 (7)	0.0013 (6)
C2	0.0285 (8)	0.0242 (8)	0.0294 (8)	−0.0015 (6)	0.0099 (7)	0.0012 (6)
C3	0.0308 (9)	0.0238 (8)	0.0319 (9)	0.0010 (7)	0.0078 (7)	0.0044 (7)
C4	0.0302 (9)	0.0195 (8)	0.0397 (9)	0.0042 (6)	0.0110 (7)	0.0021 (7)
C5	0.0245 (8)	0.0214 (8)	0.0323 (8)	−0.0037 (6)	0.0111 (7)	−0.0028 (6)
C6	0.0270 (8)	0.0204 (8)	0.0289 (8)	−0.0013 (6)	0.0104 (7)	0.0011 (6)
C7	0.0416 (10)	0.0190 (7)	0.0281 (8)	0.0031 (7)	0.0145 (7)	0.0001 (6)
C8	0.0528 (12)	0.0236 (8)	0.0350 (9)	0.0012 (8)	0.0019 (8)	−0.0025 (7)
C9	0.0333 (9)	0.0231 (8)	0.0221 (8)	−0.0012 (6)	0.0106 (7)	−0.0007 (6)
C10	0.0379 (9)	0.0196 (7)	0.0247 (8)	0.0026 (6)	0.0147 (7)	0.0008 (6)
C11	0.0357 (9)	0.0263 (8)	0.0368 (9)	0.0076 (7)	0.0193 (8)	0.0058 (7)
C12	0.0308 (9)	0.0268 (8)	0.0424 (10)	−0.0001 (7)	0.0178 (8)	0.0037 (7)
C13	0.0356 (9)	0.0212 (8)	0.0270 (8)	0.0010 (7)	0.0151 (7)	0.0018 (6)
C14	0.0318 (9)	0.0210 (7)	0.0205 (7)	0.0020 (6)	0.0102 (6)	−0.0006 (6)
C15	0.0305 (9)	0.0211 (7)	0.0241 (8)	0.0024 (6)	0.0081 (7)	0.0001 (6)
C16	0.0317 (9)	0.0311 (9)	0.0339 (9)	0.0021 (7)	0.0037 (7)	−0.0057 (7)
C17	0.0249 (8)	0.0191 (7)	0.0301 (8)	0.0000 (6)	0.0106 (7)	−0.0015 (6)
C18	0.0245 (8)	0.0210 (7)	0.0264 (8)	0.0013 (6)	0.0078 (6)	−0.0022 (6)
C19	0.0281 (8)	0.0216 (7)	0.0293 (8)	0.0009 (6)	0.0120 (7)	0.0019 (6)
C20	0.0323 (9)	0.0196 (7)	0.0328 (9)	−0.0027 (6)	0.0128 (7)	−0.0036 (6)
C21	0.0286 (8)	0.0261 (8)	0.0292 (8)	−0.0008 (6)	0.0113 (7)	−0.0050 (7)
C22	0.0255 (8)	0.0223 (8)	0.0272 (8)	0.0008 (6)	0.0110 (6)	−0.0007 (6)
C23	0.0296 (9)	0.0263 (8)	0.0250 (8)	−0.0007 (7)	0.0107 (7)	−0.0015 (6)
C24	0.0330 (9)	0.0336 (9)	0.0365 (9)	0.0020 (7)	0.0162 (8)	0.0043 (7)
C25	0.0268 (8)	0.0291 (8)	0.0229 (8)	−0.0032 (7)	0.0081 (6)	−0.0003 (6)
C26	0.0291 (9)	0.0286 (8)	0.0218 (7)	−0.0009 (7)	0.0103 (7)	0.0000 (6)
C27	0.0331 (9)	0.0277 (8)	0.0279 (8)	−0.0040 (7)	0.0091 (7)	−0.0026 (7)
C28	0.0268 (9)	0.0358 (9)	0.0363 (9)	−0.0059 (7)	0.0078 (7)	−0.0043 (7)
C29	0.0275 (9)	0.0331 (9)	0.0286 (8)	0.0027 (7)	0.0074 (7)	0.0008 (7)
C30	0.0295 (8)	0.0283 (8)	0.0201 (7)	−0.0006 (7)	0.0083 (6)	0.0012 (6)
C31	0.0299 (9)	0.0283 (8)	0.0243 (8)	0.0017 (7)	0.0076 (7)	0.0028 (6)
C32	0.0345 (9)	0.0324 (9)	0.0324 (9)	−0.0022 (7)	0.0125 (7)	0.0007 (7)
N1A	0.0351 (8)	0.0252 (7)	0.0471 (9)	0.0007 (6)	0.0135 (7)	−0.0001 (6)
N2A	0.0497 (10)	0.0269 (8)	0.0513 (10)	−0.0109 (7)	0.0114 (8)	0.0013 (7)
N3A	0.0572 (11)	0.0279 (8)	0.0475 (9)	−0.0143 (7)	0.0187 (8)	−0.0026 (7)
N4A	0.0927 (15)	0.0297 (9)	0.0385 (9)	−0.0161 (9)	0.0297 (10)	−0.0029 (7)
C1A	0.0326 (10)	0.0284 (9)	0.0562 (12)	−0.0025 (7)	0.0077 (9)	0.0045 (8)
C2A	0.0360 (10)	0.0258 (9)	0.0596 (12)	0.0015 (8)	0.0089 (9)	0.0086 (8)
C3A	0.0380 (10)	0.0268 (9)	0.0488 (11)	−0.0049 (8)	0.0138 (9)	−0.0001 (8)
C4A	0.0304 (10)	0.0352 (10)	0.0732 (15)	−0.0016 (8)	0.0149 (10)	−0.0002 (10)
C5A	0.0373 (11)	0.0284 (9)	0.0651 (13)	0.0064 (8)	0.0168 (10)	0.0014 (9)
C6A	0.0413 (11)	0.0332 (10)	0.0588 (13)	−0.0107 (9)	0.0156 (10)	−0.0014 (9)
C7A	0.0887 (18)	0.0316 (10)	0.0429 (12)	0.0000 (11)	0.0156 (12)	0.0044 (9)
C8A	0.0651 (14)	0.0350 (10)	0.0392 (11)	−0.0058 (10)	0.0077 (10)	0.0035 (8)
C9A	0.0636 (13)	0.0294 (9)	0.0247 (8)	−0.0105 (9)	0.0144 (8)	−0.0008 (7)
C10A	0.0638 (13)	0.0319 (10)	0.0436 (11)	−0.0101 (9)	0.0293 (10)	−0.0046 (8)
C11A	0.0759 (15)	0.0383 (11)	0.0483 (12)	−0.0198 (11)	0.0373 (11)	−0.0082 (9)
C12A	0.0524 (12)	0.0303 (9)	0.0299 (9)	−0.0108 (8)	0.0063 (8)	0.0018 (7)
N1B	0.0511 (10)	0.0342 (8)	0.0365 (8)	−0.0075 (7)	0.0148 (7)	−0.0088 (7)
N2B	0.0474 (10)	0.0333 (9)	0.0703 (12)	−0.0018 (8)	0.0201 (9)	−0.0022 (8)
N3B	0.0518 (11)	0.0337 (9)	0.0628 (11)	−0.0028 (8)	0.0234 (9)	0.0024 (8)
N4B	0.0536 (11)	0.0340 (9)	0.0602 (11)	0.0045 (8)	0.0133 (9)	0.0084 (8)
C1B	0.0445 (12)	0.0370 (11)	0.0728 (15)	0.0027 (9)	0.0093 (11)	−0.0009 (10)
C2B	0.0384 (11)	0.0377 (11)	0.0697 (14)	−0.0058 (9)	0.0117 (10)	0.0001 (10)
C3B	0.0405 (10)	0.0341 (9)	0.0314 (9)	−0.0019 (8)	0.0176 (8)	−0.0022 (7)
C4B	0.0386 (11)	0.0406 (11)	0.0603 (13)	0.0010 (9)	0.0206 (10)	0.0000 (9)
C5B	0.0434 (11)	0.0440 (11)	0.0528 (12)	−0.0108 (9)	0.0202 (10)	−0.0107 (9)
C6B	0.0422 (11)	0.0371 (10)	0.0442 (11)	0.0017 (8)	0.0212 (9)	0.0031 (8)
C7B	0.0489 (12)	0.0363 (10)	0.0510 (12)	−0.0029 (9)	0.0138 (10)	0.0022 (9)
C8B	0.0429 (11)	0.0376 (10)	0.0424 (11)	0.0058 (8)	0.0104 (9)	0.0031 (8)
C9B	0.0490 (12)	0.0321 (9)	0.0384 (10)	0.0009 (8)	0.0176 (9)	0.0052 (8)
C10B	0.0423 (12)	0.0409 (12)	0.0794 (16)	−0.0018 (9)	0.0145 (11)	0.0071 (11)
C11B	0.0483 (13)	0.0414 (12)	0.0886 (18)	0.0086 (10)	0.0102 (12)	0.0146 (12)
C12B	0.0489 (12)	0.0350 (10)	0.0452 (11)	0.0029 (9)	0.0184 (9)	0.0032 (8)

### Geometric parameters (Å, °)

**Table:**

O1—C3	1.3705 (19)	C26—C27	1.399 (2)
O1—H1	0.8400	C27—C28	1.386 (2)
O2—C5	1.3813 (19)	C28—C29	1.385 (2)
O2—H2	0.8400	C28—H28	0.9500
O3—C11	1.3636 (19)	C29—C30	1.392 (2)
O3—H3	0.8400	C30—C31	1.521 (2)
O4—C13	1.3800 (18)	C31—C32	1.525 (2)
O4—H4	0.8400	C31—H31	1.0000
O5—C19	1.3678 (18)	C32—H32A	0.9800
O5—H5	0.8400	C32—H32B	0.9800
O6—C21	1.3748 (18)	C32—H32C	0.9800
O6—H6	0.8400	N1A—C5A	1.328 (2)
O7—C27	1.3769 (19)	N1A—C1A	1.335 (2)
O7—H7	0.8400	N2A—C6A	1.265 (2)
O8—C29	1.382 (2)	N2A—N3A	1.414 (2)
O8—H8	0.8400	N3A—C12A	1.266 (2)
C1—C6	1.391 (2)	N4A—C7A	1.329 (3)
C1—C2	1.392 (2)	N4A—C11A	1.331 (3)
C1—H1A	0.9500	C1A—C2A	1.378 (2)
C2—C3	1.396 (2)	C1A—H1A1	0.9500
C2—C31	1.516 (2)	C2A—C3A	1.388 (3)
C3—C4	1.390 (2)	C2A—H2A	0.9500
C4—C5	1.386 (2)	C3A—C4A	1.384 (3)
C4—H4A	0.9500	C3A—C6A	1.475 (2)
C5—C6	1.396 (2)	C4A—C5A	1.379 (3)
C6—C7	1.516 (2)	C4A—H4A1	0.9500
C7—C10	1.517 (2)	C5A—H5A	0.9500
C7—C8	1.525 (2)	C6A—H6A	0.9500
C7—H7A	1.0000	C7A—C8A	1.393 (3)
C8—H8A	0.9800	C7A—H7A1	0.9500
C8—H8B	0.9800	C8A—C9A	1.375 (3)
C8—H8C	0.9800	C8A—H8A1	0.9500
C9—C10	1.391 (2)	C9A—C10A	1.389 (3)
C9—C14	1.398 (2)	C9A—C12A	1.475 (2)
C9—H9	0.9500	C10A—C11A	1.383 (2)
C10—C11	1.397 (2)	C10A—H10A	0.9500
C11—C12	1.387 (2)	C11A—H11A	0.9500
C12—C13	1.386 (2)	C12A—H12A	0.9500
C12—H12	0.9500	N1B—C5B	1.323 (3)
C13—C14	1.393 (2)	N1B—C1B	1.328 (3)
C14—C15	1.527 (2)	N2B—C6B	1.252 (2)
C15—C18	1.526 (2)	N2B—N3B	1.408 (2)
C15—C16	1.530 (2)	N3B—C12B	1.273 (3)
C15—H15	1.0000	N4B—C7B	1.322 (3)
C16—H16A	0.9800	N4B—C11B	1.335 (3)
C16—H16B	0.9800	C1B—C2B	1.390 (3)
C16—H16C	0.9800	C1B—H1B	0.9500
C17—C22	1.394 (2)	C2B—C3B	1.372 (3)
C17—C18	1.395 (2)	C2B—H2B	0.9500
C17—H17	0.9500	C3B—C4B	1.374 (3)
C18—C19	1.398 (2)	C3B—C6B	1.471 (2)
C19—C20	1.391 (2)	C4B—C5B	1.379 (3)
C20—C21	1.389 (2)	C4B—H4B	0.9500
C20—H20	0.9500	C5B—H5B	0.9500
C21—C22	1.398 (2)	C6B—H6B	0.9500
C22—C23	1.531 (2)	C7B—C8B	1.384 (3)
C23—C26	1.522 (2)	C7B—H7B	0.9500
C23—C24	1.531 (2)	C8B—C9B	1.379 (3)
C23—H23	1.0000	C8B—H8B1	0.9500
C24—H24A	0.9800	C9B—C10B	1.389 (3)
C24—H24B	0.9800	C9B—C12B	1.472 (3)
C24—H24C	0.9800	C10B—C11B	1.380 (3)
C25—C30	1.392 (2)	C10B—H10B	0.9500
C25—C26	1.400 (2)	C11B—H11B	0.9500
C25—H25	0.9500	C12B—H12B	0.9500
			
C3—O1—H1	109.5	O7—C27—C28	115.51 (15)
C5—O2—H2	109.5	O7—C27—C26	123.57 (15)
C11—O3—H3	109.5	C28—C27—C26	120.91 (15)
C13—O4—H4	109.5	C29—C28—C27	120.04 (15)
C19—O5—H5	109.5	C29—C28—H28	120.0
C21—O6—H6	109.5	C27—C28—H28	120.0
C27—O7—H7	109.5	O8—C29—C28	120.55 (15)
C29—O8—H8	109.5	O8—C29—C30	117.98 (15)
C6—C1—C2	123.60 (14)	C28—C29—C30	121.40 (15)
C6—C1—H1A	118.2	C25—C30—C29	116.94 (15)
C2—C1—H1A	118.2	C25—C30—C31	125.33 (14)
C1—C2—C3	117.53 (14)	C29—C30—C31	117.72 (14)
C1—C2—C31	121.81 (14)	C2—C31—C30	112.53 (13)
C3—C2—C31	120.54 (14)	C2—C31—C32	109.18 (13)
O1—C3—C4	122.11 (14)	C30—C31—C32	114.24 (13)
O1—C3—C2	117.35 (14)	C2—C31—H31	106.8
C4—C3—C2	120.52 (15)	C30—C31—H31	106.8
C5—C4—C3	120.16 (14)	C32—C31—H31	106.8
C5—C4—H4A	119.9	C31—C32—H32A	109.5
C3—C4—H4A	119.9	C31—C32—H32B	109.5
O2—C5—C4	121.49 (14)	H32A—C32—H32B	109.5
O2—C5—C6	117.28 (14)	C31—C32—H32C	109.5
C4—C5—C6	121.20 (14)	H32A—C32—H32C	109.5
C1—C6—C5	116.98 (14)	H32B—C32—H32C	109.5
C1—C6—C7	122.22 (14)	C5A—N1A—C1A	117.15 (15)
C5—C6—C7	120.62 (14)	C6A—N2A—N3A	112.75 (16)
C6—C7—C10	113.06 (13)	C12A—N3A—N2A	110.74 (16)
C6—C7—C8	108.58 (13)	C7A—N4A—C11A	117.26 (17)
C10—C7—C8	114.46 (14)	N1A—C1A—C2A	123.46 (17)
C6—C7—H7A	106.8	N1A—C1A—H1A1	118.3
C10—C7—H7A	106.8	C2A—C1A—H1A1	118.3
C8—C7—H7A	106.8	C1A—C2A—C3A	118.73 (17)
C7—C8—H8A	109.5	C1A—C2A—H2A	120.6
C7—C8—H8B	109.5	C3A—C2A—H2A	120.6
H8A—C8—H8B	109.5	C4A—C3A—C2A	118.13 (16)
C7—C8—H8C	109.5	C4A—C3A—C6A	120.06 (17)
H8A—C8—H8C	109.5	C2A—C3A—C6A	121.80 (17)
H8B—C8—H8C	109.5	C5A—C4A—C3A	118.74 (18)
C10—C9—C14	123.40 (15)	C5A—C4A—H4A1	120.6
C10—C9—H9	118.3	C3A—C4A—H4A1	120.6
C14—C9—H9	118.3	N1A—C5A—C4A	123.72 (17)
C9—C10—C11	117.32 (14)	N1A—C5A—H5A	118.1
C9—C10—C7	124.67 (15)	C4A—C5A—H5A	118.1
C11—C10—C7	118.00 (14)	N2A—C6A—C3A	120.58 (18)
O3—C11—C12	122.67 (15)	N2A—C6A—H6A	119.7
O3—C11—C10	116.38 (14)	C3A—C6A—H6A	119.7
C12—C11—C10	120.93 (15)	N4A—C7A—C8A	123.6 (2)
C13—C12—C11	119.92 (16)	N4A—C7A—H7A1	118.2
C13—C12—H12	120.0	C8A—C7A—H7A1	118.2
C11—C12—H12	120.0	C9A—C8A—C7A	118.6 (2)
O4—C13—C12	115.10 (14)	C9A—C8A—H8A1	120.7
O4—C13—C14	123.51 (14)	C7A—C8A—H8A1	120.7
C12—C13—C14	121.38 (14)	C8A—C9A—C10A	118.37 (17)
C13—C14—C9	116.94 (14)	C8A—C9A—C12A	120.24 (19)
C13—C14—C15	119.66 (13)	C10A—C9A—C12A	121.39 (18)
C9—C14—C15	123.33 (14)	C11A—C10A—C9A	118.8 (2)
C18—C15—C14	108.28 (12)	C11A—C10A—H10A	120.6
C18—C15—C16	111.73 (13)	C9A—C10A—H10A	120.6
C14—C15—C16	114.39 (13)	N4A—C11A—C10A	123.4 (2)
C18—C15—H15	107.4	N4A—C11A—H11A	118.3
C14—C15—H15	107.4	C10A—C11A—H11A	118.3
C16—C15—H15	107.4	N3A—C12A—C9A	121.26 (19)
C15—C16—H16A	109.5	N3A—C12A—H12A	119.4
C15—C16—H16B	109.5	C9A—C12A—H12A	119.4
H16A—C16—H16B	109.5	C5B—N1B—C1B	116.28 (17)
C15—C16—H16C	109.5	C6B—N2B—N3B	112.47 (17)
H16A—C16—H16C	109.5	C12B—N3B—N2B	112.35 (17)
H16B—C16—H16C	109.5	C7B—N4B—C11B	116.87 (18)
C22—C17—C18	123.82 (14)	N1B—C1B—C2B	124.14 (19)
C22—C17—H17	118.1	N1B—C1B—H1B	117.9
C18—C17—H17	118.1	C2B—C1B—H1B	117.9
C17—C18—C19	117.11 (14)	C3B—C2B—C1B	118.73 (19)
C17—C18—C15	121.42 (13)	C3B—C2B—H2B	120.6
C19—C18—C15	121.26 (14)	C1B—C2B—H2B	120.6
O5—C19—C20	121.14 (13)	C2B—C3B—C4B	117.38 (17)
O5—C19—C18	117.98 (13)	C2B—C3B—C6B	123.39 (17)
C20—C19—C18	120.83 (14)	C4B—C3B—C6B	119.17 (17)
C21—C20—C19	120.21 (14)	C3B—C4B—C5B	119.97 (19)
C21—C20—H20	119.9	C3B—C4B—H4B	120.0
C19—C20—H20	119.9	C5B—C4B—H4B	120.0
O6—C21—C20	121.00 (14)	N1B—C5B—C4B	123.49 (19)
O6—C21—C22	117.97 (14)	N1B—C5B—H5B	118.3
C20—C21—C22	121.00 (14)	C4B—C5B—H5B	118.3
C17—C22—C21	116.97 (14)	N2B—C6B—C3B	123.24 (18)
C17—C22—C23	121.08 (13)	N2B—C6B—H6B	118.4
C21—C22—C23	121.89 (14)	C3B—C6B—H6B	118.4
C26—C23—C22	110.34 (12)	N4B—C7B—C8B	123.66 (19)
C26—C23—C24	114.45 (13)	N4B—C7B—H7B	118.2
C22—C23—C24	110.78 (13)	C8B—C7B—H7B	118.2
C26—C23—H23	107.0	C9B—C8B—C7B	119.09 (18)
C22—C23—H23	107.0	C9B—C8B—H8B1	120.5
C24—C23—H23	107.0	C7B—C8B—H8B1	120.5
C23—C24—H24A	109.5	C8B—C9B—C10B	118.01 (18)
C23—C24—H24B	109.5	C8B—C9B—C12B	120.71 (18)
H24A—C24—H24B	109.5	C10B—C9B—C12B	121.27 (18)
C23—C24—H24C	109.5	C11B—C10B—C9B	118.4 (2)
H24A—C24—H24C	109.5	C11B—C10B—H10B	120.8
H24B—C24—H24C	109.5	C9B—C10B—H10B	120.8
C30—C25—C26	123.61 (15)	N4B—C11B—C10B	123.9 (2)
C30—C25—H25	118.2	N4B—C11B—H11B	118.0
C26—C25—H25	118.2	C10B—C11B—H11B	118.0
C27—C26—C25	116.90 (14)	N3B—C12B—C9B	119.98 (18)
C27—C26—C23	119.19 (14)	N3B—C12B—H12B	120.0
C25—C26—C23	123.90 (14)	C9B—C12B—H12B	120.0

### Hydrogen-bond geometry (Å, °)

**Table:**

*D*—H···*A*	*D*—H	H···*A*	*D*···*A*	*D*—H···*A*
O1—H1···N4B	0.84	1.95	2.765 (2)	162
O2—H2···N1A	0.84	1.87	2.6986 (17)	167
O3—H3···O2^i^	0.84	1.99	2.8297 (17)	172
O4—H4···O5	0.84	2.15	2.9265 (17)	153
O5—H5···N4A^ii^	0.84	1.90	2.7338 (19)	175
O6—H6···N1B^ii^	0.84	1.95	2.7855 (19)	176
O7—H7···O6	0.84	2.14	2.9528 (18)	163
O8—H8···N2B^iii^	0.84	2.11	2.947 (2)	174

Symmetry codes: (i) −*x*, −*y*, −*z*; (ii) *x*, *y*−1, *z*; (iii) −*x*, *y*−1/2, −*z*−1/2.
